# Effect of remote ischaemic conditioning on left ventricular function in ST-segment elevation myocardial infarction patients: The CONDI-2 echocardiographic sub-study

**DOI:** 10.3389/fcvm.2022.1054142

**Published:** 2023-01-25

**Authors:** Gregory Wood, Pia Hedegaard Johnsen, Anders Lehmann Dahl Pedersen, Christian Alcaraz Frederiksen, Steen Hvitfeldt Poulsen, Hans Erik Bøtker, Won Yong Kim

**Affiliations:** ^1^Department of Cardiology, Aarhus University Hospital, Aarhus, Denmark; ^2^Department of Clinical Medicine, Aarhus University Hospital, Aarhus, Denmark

**Keywords:** ST-elevation myocardial infarction, remote ischaemic conditioning, echocardiography, CONDI-2/ERIC-PPCI, left ventricular function

## Abstract

**Background:**

Remote ischaemic conditioning (RIC) applied to the arm by inflation and deflation of a pneumatic cuff has been shown to reduce myocardial infarct size in patients with ST-elevation myocardial infarction undergoing primary percutaneous coronary intervention (PPCI). However, the effect of RIC on left ventricular ejection fraction (LVEF) following infarct healing remains unknown.

**Objective:**

To investigate whether RIC applied in the ambulance before PPCI can improve left ventricular (LV) function in STEMI patients 3 months following infarction.

**Methods:**

Echocardiography was performed in a total of 694 patients from the CONDI-2 study a median of 112 days (IQR 63) after the initial admission. LVEF and LV end-diastolic and end-systolic volumes were calculated using the modified Simpsons biplane method of disks. LV global longitudinal strain (GLS) was estimated using 2-dimensional cine-loops with a frame rate > 55 frames/second, measured in the three standard apical views.

**Results:**

There was no difference in the measured echocardiographic parameters in the RIC group as compared to the control group, including LV EF, LV GLS, tricuspid annular plane systolic excursion or left ventricular volumes. In the control group, 32% had an ejection fraction < 50% compared to 37% in the RIC group (p = 0.129).

**Conclusion:**

In this largest to date randomized imaging study of RIC, RIC as an adjunct to PPCI was not associated with a change in echocardiographic measures of cardiac function compared to standard PPCI alone.

## Introduction

Remote ischemic conditioning (RIC) by 5-min cycles of ischemia and reperfusion has been proposed as an adjunct to primary percutaneous coronary intervention (PPCI) in patients with ST-elevation myocardial infarction (STEMI). This was initially demonstrated in animal models (1, 2), then extensively investigated in patients, demonstrating an increase in myocardial salvage in patients with STEMI in smaller, single-centre studies (3–6). However, the international randomized multi-centre CONDI-2/ERIC-PPCI trial, including 5,401 STEMI patients, demonstrated no reduction in the rates of cardiac death or hospitalization for heart failure at 12 months by RIC (7).

A prospectively pre-specified sub-analysis of the CONDI-2/ERIC-PPCI trial, assessing changes in cardiovascular magnetic resonance (CMR) parameters, did not demonstrate statistically significant differences between the control and intervention groups, however an improvement in Left Ventricular Ejection Fraction (LVEF) and Microvascular Obstruction (MVO) in the RIC group was demonstrated on the acute scan when specifically assessing Left Anterior Descending (LAD) STEMI (8). The results are consistent with previous findings from our research group, whereby a modest short-term improvement in left ventricular (LV) function was demonstrated in high-risk patients treated with RIC (9). A recent study by Chen et al. including 60 patients demonstrated no improvement in LVEF. However, a marginal improvement in Global Longitudinal Strain (GLS) was shown (10) 1-month post-infarct as compared to baseline.

As such, conclusive evidence regarding the effects of RIC on echocardiographic variables of cardiac function following healed STEMI are required. In this pre-specified CONDI-2 echocardiographic single-center sub-study, we investigated the effect of RIC on LVEF and global longitudinal strain using echocardiography at 3 month follow-up.

## Materials and methods

The CONDI-2 study received ethical approval from regional and national health service research ethics committees and was conducted in accordance with the principles of good clinical practice. All participants provided written informed consent before randomization. This echocardiographic sub-study was planned prior to commencement of the multi-centre study.

In the CONDI-2 study, patients with chest pain were included if they were older than 18 years of age, had ST-segment elevation on ECG, and were eligible for PPCI (7). Patients were randomly allocated (1:1) to a remote ischaemic conditioning group or a control group through a web-based clinical trial support system accessible 24 h a day (TrialPartner, Aarhus, Denmark).

RIC was administered in the ambulance before PPCI in patients randomized to the RIC group. RIC was performed using an automated cuff (CellAegis AutoRIC, Toronto, Ontario, Canada) on the upper arm. RIC comprised of 4 consecutive periods of inflation and deflation of the cuff from 0 mmHg and 200 mmHg, lasting two consecutive periods of 5 minutes each. Patients randomized to control received standard therapy.

All patients enrolled in the CONDI-2 echocardiographic sub-study underwent echocardiographic assessment according to guidelines (11). A GE Vivid E9 or E95 ultrasound machine (GE healthcare, Horten, Norway) with a standard phased array transducer was used. Offline analysis was performed using Echopac version 203 (PC SW-only, GE Healthcare, Milwaukee, Wisconsin, USA).

LVEF and LV end-diastolic (LV EDV) and end-systolic (LV ESV) volumes were calculated using the modified Simpsons biplane method of disks. LV global longitudinal strain (GLS) was estimated using 2-dimensional cine-loops with a frame rate > 55 frames/second, measured in the three standard apical views. The tracking algorithm automatically traced the endocardial border and was manually adjusted as appropriate. The software automatically generated a 17-segment bullseye plot, with the presented values being an average of all 17 segments. Tricuspid Annular Plane Systolic Excursion (TAPSE) was measured using M-mode in the apical 4-chamber view. All echocardiographic analysis was performed in a blinded manner by two investigators experienced in echocardiography. The first 50 analyses were performed in co-operation by the two experts in order to establish a consensus.

High-sensitivity troponin T was measured at admission and 6-8 hours, 24 hours and 48-72 hours following admission, as described in the study protocol (7). The peak value was used as a surrogate marker of infarct size.

## Results

A total of 694 patients from Aarhus University Hospital were included between January 2014 and March 2018. Baseline demographics did not differ between the RIC and control groups (Table 1). Peak high-sensitivity troponin T did not differ between the RIC and the control groups. Echocardiography was performed a median of 110 days (IQR 110-149) after the initial admission.

**TABLE 1:** Demographics at follow-up following myocardial infarction.

Variable	Total (*n* = 715)	Control (*n* = 355)	RIC (*n* = 360)	*P*-value
Age, years	63 ± 12	63 ± 12	64 ± 12	0.35
Male gender	77% (551)	77% (274)	77% (277)	0.94
BMI, kg/m^2^	27 ± 4	27 ± 4	27 ± 5	0.78
SBP	132 ± 25	133 ± 25	131 ± 25	0.33
DBP	72 ± 14	72 ± 14	72 ± 13	0.68
Hypertension	36% (260)	34% (122)	38% (138)	0.27
Previous MI	9% (64)	9% (31)	9% (33)	0.84
Hypercholesterolaemia	22% (159)	21% (75)	23% (84)	0.48
Diabetes	8% (56)	6% (22)	9% (34)	0.11
Statins	24% (171)	23% (82)	25% (89)	0.53
Beta-blocker	12% (87)	10% (37)	14% (50)	0.16
ACE-inhibitor	11% (80)	10% (35)	13% (45)	0.16
ARB	13% (94)	12% (42)	14% (52)	0.30
Aspirin	15% (108)	14% (49)	16% (59)	0.33
Clopidogrel	2% (15)	2% (8)	2% (7)	0.77
Ticagrelor	0.3% (2)	0.0% (0)	0.6% (2)	0.16
Peak TnT, ng/l	1523 (568–3000)	1426 (583–2613)	1575 (506–3369)	0.84
Symptom to balloon time, minutes	161 (117–245)	161 (119–254)	161 (116–240)	0.86
Culprit vessel – LAD – Circumflex – RCA – Missing	40% 13% 33% 15%	39% 15% 32% 14%	41% 10% 34% 15%	0.31

Data are presented as mean ± standard deviation or median (interquartile range) or percentages (*n*). BMI, body mass index; SBP, systolic blood pressure; DBP, diastolic blood pressure; MI, myocardial infarction; ACE, angiotensin-converting enzyme; ARB, angiotensin II receptor blocker; TnT, high-sensitivity troponin T; LAD, left anterior descending artery; RCA, right coronary artery.

LVEF was no different in the RIC group than in the control group (53.4% [48-58] vs. 52.8% [48-58], *p* = 0.30) (Table 2). In the control group, 32% had an LVEF < 50% compared to 37% in the RIC group (*p* = 0.129). Neither LV volumes, LV GLS or (TAPSE) differed significantly between the 2 groups.

**TABLE 2:** Echocardiography results at follow-up following myocardial infarction.

Variable	Total (*n* = 715)	Control (*n* = 355)	RIC (*n* = 360)	*P*-value
Days to TTE	110 (86–149)	112 (87–155)	108 (86–147)	0.50
LV EF	52.9 (48–58)	53.4 (48–58)	52.8 (48–58)	0.30
**LV EF – culprit vessel**				
LAD	52.1 (46–58)	51.9 (46–58)	52.2 (46–58)	0.98
CX	53.7 (49–58)	53.8 (49–58)	52.9 (47–58)	0.95
RCA	52.9 (49–57)	53.4 (49–58)	52.9 (49–56)	0.17
LV EDV	105 (87–130)	105 (87–131)	105 (86–129)	0.66
LV ESV	49 (39–63)	49 (39–63)	50 (39–63)	0.78
LV SV	56 ± 16	57 ± 16	56 ± 16	0.32
LV GLS	−17.1 (−18;−15)	−17.1 (−18;−15)	−17.1 (−18;−15)	0.19
TAPSE	2.2 ± 0.4	2.2 ± 0.4	2.2 ± 0.4	0.34

Data are presented as mean ± standard deviation or median (interquartile range). TTE, transthoracic echocardiography; LV, left ventricle; EF, ejection fraction; LAD, left anterior descending artery; CX, Circumflex; RCA, right coronary artery; EDV, end-diastolic volume; ESV, end-systolic volume; SV, stroke volume; GLS, global longitudinal strain; TAPSE, tricuspid annular plane systolic excursion.

**FIGURE 1:**
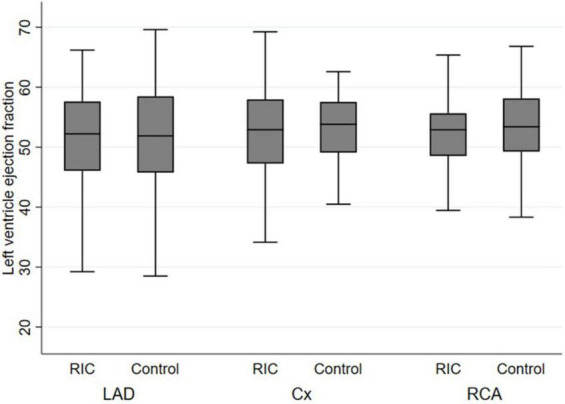
Left ventricular ejection fraction stratified by culprit vessel. LAD, left anterior descending artery; CX, circumflex artery; RCA, right coronary artery; RIC, remote ischaemic conditioning.

Subgroup analysis did not show significant differences with respect to coronary territories (Figure 1 and Table 2). Although not statistically significantly different, LVEF was higher in women than in men (53.7% [49–59] vs. 52.7% [48–58], *p* = 0.14).

## Discussion

Our study demonstrated no difference in measured echocardiographic variables within the RIC treatment group as compared to controls. In the cardiac magnetic resonance (CMR) sub-study of 169 patients in the CONDI-2/ERIC-PPCI trial, RIC did not reduce myocardial infarct size or improve LVEF at six months when assessed as a whole cohort. However, a difference was observed when LAD STEMI patients alone were compared on the acute scans (8). This observation was not seen with our data, as LVEF in individuals with the LAD as the culprit vessel did not differ (Figure 1), which was also the case with the other culprit vessels.

We did not find any difference in GLS between the RIC and control groups. Previous data has suggested that GLS in the RIC group improves to a greater extent than that of the control group 1 month following STEMI (10). However, in contrast to our study, RIC was not initiated before reperfusion but rather within 24 hours after PPCI and repeated daily for one week. Similarly, another echocardiographic study demonstrated that late repetitive RIC following PPCI improved longitudinal strain in the infarct-related segments and led to an attenuated increase in circumferential strain in remote segments (12). These results suggest that RIC may prevent pathological remodeling in the immediate post-infarct period, beyond an immediate cardio-protective effect during the ischaemia-reperfusion phase. However, the reported GLS differences are small, so further research may be required to determine the clinical significance of these improvements in GLS.

Interestingly, peak high-sensitivity Troponin T was similar between groups. This supports the neutral findings of the imaging parameters.

These findings further support the neutral results regarding clinical endpoints observed from the multi-centre CONDI-2/ERIC-PPCI trial, indicating that RIC may not be effective as an adjunct to standard treatment for STEMI. This echocardiographic sub-study provides a good approximation of the participants of the CONDI-2/ERIC-PPCI (7), as the baseline demographics and treatment broadly reflect that of the main clinical study, albeit the proportion of those receiving aspirin, ticagrelor and clopidogrel is slightly less in our study (Table 1). However, it should also be noted that it is unknown whether an underlying difference may have existed prior to STEMI, although randomized allocation was performed to mitigate differences between the RIC and control groups. Furthermore, there may exist other uncontrolled for confounding factors which could have influenced the measures of LV function.

Other applications of RIC are currently being investigated. In a recent experimental study, repetitive remote ischaemic preconditioning was shown to ameliorate anthracycline-induced cardiotoxicity resulting in significantly higher long-term LV EF and less cardiac fibrosis (13). This approach is now being tested in the RESILIENCE (remote ischemic conditioning in lymphoma patients receiving anthracyclines) trial (14) which is a large randomized clinical multicenter trial to evaluate the efficacy and safety of remote ischaemic preconditioning in Non-Hodgkin Lymphoma patients receiving anthracyclines.

In conclusion, in this largest to date randomized imaging study of RIC, there were no differences in measured echocardiographic variables by RIC as an adjunct to PPCI compared to standard PPCI alone. These results further support our previous findings that RIC did not improve clinical outcomes at 12 months in patients with STEMI undergoing PPCI.

## Data availability statement

The raw data supporting the conclusions of this article will be made available by the authors, without undue reservation.

## Ethics statement

The studies involving human participants were reviewed and approved by Central Jutland Regional Committee on Health Research Ethics. The patients/participants provided their written informed consent to participate in this study.

## Author contributions

HB, SP, and WK were responsible for the conception and design of the study. PJ and CF performed the data acquisition. GW, PJ, AP, and WK performed the data analysis and interpretation. GW and WK wrote the manuscript. All authors reviewed and approved the manuscript prior to submission.
